# Intercultural Communication between Long-Stay Immigrants and Catalan Primary Care Nurses: A Qualitative Approach to Rebalancing Power

**DOI:** 10.3390/ijerph18062851

**Published:** 2021-03-11

**Authors:** Francesc Ramos-Roure, Maria Feijoo-Cid, Josep Maria Manresa-Dominguez, Jordi Segura-Bernal, Rosa García-Sierra, Maria Isabel Fernández-Cano, Pere Toran-Monserrat

**Affiliations:** 1 Research Support Unit Metropolitana Nord, Primary Care Research Institut Jordi Gol (IDIAPJGol), 08303 Barcelona, Spain; rourenet@gmail.com (F.R.-R.); jmanresa@idiapjgol.info (J.M.M.-D.); rgarciasi.mn.ics@gencat.cat (R.G.-S.); ptoran.bnm.ics@gencat.cat (P.T.-M.); 2 Grup de REcerca Multidisciplinar en SAlut i Societat (GREMSAS), (2017 SGR 917), 08007 Barcelona, Spain; mariaisabel.fernandezc@uab.cat; 3 Department of Medicine, Faculty of Medicine, Universitat Autónoma de Barcelona, 08193 Bellaterra, Spain; 4 Department of Nursing, Faculty of Medicine, Universitat Autónoma de Barcelona, 08193 Bellaterra, Spain; 5 Faculty of Psychology, Education Sciences and Sport, Ramon Llull University, 08022 Barcelona, Spain; jordisb@blanquerna.url.edu; 6 Department of Medicine, Faculty of Medicine, Universitat de Girona, 17071 Girona, Spain

**Keywords:** intercultural competence, intercultural communication, long-stay immigrant, nurse-patient relations, primary care, cultural safety

## Abstract

There is a gap between the preferences of immigrant patients and their experiences with intercultural communication. This study aims to explore the experiences and perspectives of long-stay immigrants on intercultural communication in encounters with primary care (PC) nurses. Participants were selected by purposive sampling at the Maresme Primary Care Center. A focus group and five in-depth interviews with long-stay immigrants from eight countries were carried out. Data collection was guided by a script previously validated by a group of experts. We conducted a qualitative analysis following Charmaz’s approach, and data saturation was reached with 11 patients (one focus group and five interviews). Long-stay immigrants would like closer and more personalized communication exchanges with greater humanity, as well as polite and respectful manners as they perceive signs of an asymmetrical care relationship. Those who had negative communication experiences tried to justify some of the behaviors as a result of having free access to public health services. This is one of the few existing studies from the point of view of long-stay immigrants. Achieving effective intercultural communication requires a process of self-reflection, awareness-raising and commitment, both on a personal and institutional level, to eliminate the asymmetry in the nurse-patient relationship. Nurses should be trained in person-centered intercultural communication.

## 1. Introduction

Immigration is a global phenomenon entailing socio-demographic changes and greater cultural diversity in host societies. People immigrate mainly in search of better living conditions, settling down in the hosting country for a long period of time or permanently [1]. In Spain, the immigration boom started at the turn of the millennium [2] but in Catalonia, it has been continuously on the rise since the 1980s [3]. Until 2012, access to health care in Spain involved uncomplicated administrative procedures, was free of charge and guaranteed emergency care for all citizens [4]. The global financial crisis led to restrictive measures that resulted in the 2012 reform, which put an end to universal access to healthcare: now, access to the Spanish National Health System (NHS) was limited to those covered by health insurance, thus excluding undocumented immigrants, with the exception of minors, pregnant women and emergencies [5]. In 2018, new legislation (RDL 7/201812) was passed aimed at reestablishing the universality of the NHS but still maintaining access requirements. As a result, it does not ensure coverage for the entire population, but does include the aforementioned exceptions [6]. 

The health needs, expectations and previous care experiences of immigrant communities often differ from those of the local population. While the health status of incoming immigrants is often better than that of the native population [7], the conditions they face in the host country (e.g., social isolation, discrimination, cultural changes, separation from family, limited income) are often considerably different from their expectations, which can lead to unhealthy lifestyles, stress, anxiety, and depression [8,9]. The poor health of this population is the result of their limited socio-economic and political power, and scanty health-related resources [10]. The literature shows that the main barriers they face when accessing the healthcare system are related to language, culture and communication [11,12,13,14].

### 1.1. Intercultural Competence in Healthcare

This scenario poses challenges to the cultural competence of healthcare professionals, organizations and the healthcare system in the provision of quality care and the reduction of health inequalities in groups culturally different to the professionals providing health services [15]. Health science literature talks about cultural competence while other disciplines prefer the term “intercultural competence” to emphasize its bidirectional and interactive nature [16]. However, too often this bidirectional nature is left unexplored, as intercultural competence is analyzed and put into practice at the individual level, usually by health professionals, based on the understanding that competence depends on individual knowledge of rules/prescriptions for what to do and how to proceed in specific circumstances, groups, times and places [17]. Although there is no consensus on the definition, several authors have described it as “the capacity to effectively manage interactions with individuals, families and communities from diverse cultural contexts,” which is a key element to overcoming barriers and providing optimal, equitable care [17,18]. Intercultural competence is interaction and involves a continuous learning process in which intercultural communication is one of the pillars of healthcare for immigrants [19,20]. Intercultural competence and intercultural communication are used interchangeably in various disciplines [16] since competence is demonstrated in the communication process and the relational act. 

Intercultural communication is the interaction between healthcare professionals and patients from different cultural backgrounds in order to reach understanding, build a shared reality and establish a satisfactory relationship [21]. It requires verbal and non-verbal communication skills, cultural sensitivity, constructive emotional expression, cultural knowledge and adaptability [22]. This definition goes beyond the individual, contemplating the relationship as a thing of two, involving social actors with the capacity to “build a shared reality” [23]. Spitzberg and Changnon introduce the notion of “relationality” in intercultural communication, i.e., they are interested in understanding the processes that individuals engage in to develop their competences and in how people manage intercultural interactions. However, the dialectical approach proposed by Martin goes beyond that, placing individual competence traits in relational tension with the motivations, knowledge and skills of others and wider organizational and cultural contexts [21]. In the dialectical approach, the context is critical as it is a fluid and dynamic space continuously being reshaped by local and global forces [21]. Martin highlights that power relations inherent to any intercultural encounter exist in every context [21]. 

### 1.2. Immigrant Acculturation

From their arrival to the host country, immigrants are immersed in a linear, dynamic process of acculturation aimed at developing the cultural, social and communicative competences that allow them to adapt to a new living environment and type of social interaction [24,25] In short, the acculturation process is aimed at becoming interculturally competent. Language proficiency is a key element in the acculturation of these groups and is associated, in the case of non-Spanish speakers, with the duration of residence [26]. Obviously, the amount of time spent in the host country influences not only the language proficiency of immigrants but also their health status, relationships with healthcare professionals, and access to and use of the healthcare system and prevention services [27,28]. For example, in the case of pregnant immigrant women, the acculturation process reduces the risk of miscarriage and improves communication with and access to healthcare services [29]. 

Despite the fact that immigrant patients prefer clear, welcoming, respectful intercultural communication and personalized interaction in intercultural encounters [19,20,30,31], several barriers have been identified in the primary care setting, such as language [11], poor communication skills of the professionals involved (no active listening, excessive use of technicalities, asymmetric conversation), and difficulties in creating an atmosphere of trust and respect [31]. There are several key barriers to intercultural competence, many of which are not just cultural. These barriers may also respond to other factors, whether personal, social or structural [32,33], and in the case of the healthcare system, are rooted in the existing power dynamics and hierarchical structures. For example, asymmetrical conversations and the unequal relationships between the nurse and immigrant patient reflect the power dynamics embedded in the healthcare system. The current model of care is ruled by the hegemonic medical model and strongly shaped by power dynamics and very hierarchical structures. In New Zealand, analysis of this asymmetry in care relationships with the indigenous Maori population has led to the concept of “cultural safety” [15], which is related to power dynamics and the imposition of the dominant biomedical culture and its negative effects on communication, caregiving, shared decision-making, and trust. Another example of a barrier not directly linked to cultural aspects is the underuse of the health services, which may well be due to difficulties accessing it (e.g., lack of residence permit) or poor health literacy on the functioning of the healthcare system [32].

### 1.3. Research Gap

Most publications on intercultural communication focus on general immigrant populations [20,31,34,35] or the period immediately after arrival in especially vulnerable populations, such as refugees or undocumented immigrants [36,37]. There is evidence of difficulties in the continuity of care provided to immigrant patients, adherence to treatment, perception of health status, degree of satisfaction with the care provided, and trust in the healthcare system [38,39,40]. A recent literature review points to the need for qualitative studies from the point of view of immigrant patients and underlines the importance of improving immigrant patient-provider relationships in primary care [41]. However, research on intercultural communication from the perspective of linguistically competent long-stay immigrants with long acculturation processes is scarce. 

In light of all this, we took a qualitative methodological approach, using in-depth interviews and a focus group of long-stay immigrants to explore intercultural communication from their perspective. The results of this study will allow us to assess whether communication barriers are overcome or maintained throughout the acculturation process and whether emerging obstacles are individual or structural in nature, and thus affect the non-immigrant population as well. In short, the aim of this study is to explore how long-stay immigrants experience and perceive intercultural communication in encounters with primary care nurses.

## 2. Materials and Methods

### 2.1. Study Design

A qualitative socio-constructivist study was conducted to explore how long-stay immigrants experience and perceive intercultural communication with primary care nurses [42]. This theoretical approach is based on the need to understand how people construct and interpret social reality in their daily lives (in this study, long-stay immigrants) [42]. The constructivist approach does not support the idea that theories are discovered but rather that the world under study must be interpretatively depicted as interviewee and researcher engage together in the process of constructing realities [43]. Since the objective was to understand the perspectives of long-stay immigrants, the data collection method consisted of one focus group and five face-to-face, in-depth interviews.

### 2.2. Study Setting

The study was held at the Maresme Primary Care Center, in Mataró (Catalonia, Spain), which provides services in primary care, medical specialties, radiology, rehabilitation and primary care emergency, covering several basic health areas in the county of Maresme. Mataró, the largest city in the county, has 124,280 registered inhabitants, of which 11.77% to 16.01% are immigrants, depending on the neighborhood [44].

### 2.3. Ethical Considerations

The research project was approved by the primary care center’s ethics committee. Before beginning the interviews and focus group, participants were reminded of the objectives of the study that audio recordings would be taken of the sessions. Participants were reassured that confidentiality would be maintained at all times and that the data would be processed without participants being identified. They were also reminded of their right to abandon the project at any time. Immigrant patients participated voluntarily and gave their informed consent.

### 2.4. Participants and Sampling

Participants were selected by purposive sampling based on the following criteria: (1) over 25 years old (the 25 to 44 age bracket is the largest in southern Europe while the 15 to 24 bracket is less extensive [45]); (2) immigrated to Spain for economic reasons, since this is the main reason for immigration and it is easier to access these groups than political or war refugees; (3) long-stay immigrant (residing in Spain for more than six years); (4) fluent in Catalan or Spanish to allow for fluid communication; (5) attended a primary care (PC) center at least once during the last year (the GP visit rate is higher among immigrants than the native Spanish population, specifically in patients from the Maghreb, the rest of Africa and Latin America, once they have had an initial visit [46]); and (6) belongs to one of the larger immigrant populations in Catalonia (East Asia, the Andean states, Hindustan, Sub-Saharan Africa, or the Maghreb region) (Table 1 and Table 2). This last criterion was included to facilitate recruitment since in our setting there is evidence pointing to recruitment difficulties and limited knowledge of Moroccan, Pakistani and Chinese populations [47]. Individuals with physical, psychological, or sensory limitations that would interfere with verbal expression were excluded, as well as those who did not understand the project or the informed consent. Maximum heterogeneity was pursued regarding sex (men and women), age (25–45, 46–60, over 60), years of residence in Spain (6–10, 11–20, over 20 years), as well as level of education and country of origin in order to gather diverse perspectives about the topics under discussion. 

Participants were recruited by multiple parties: healthcare workers, cultural mediators and the first author. In qualitative research methods, using a combination of sampling methods is the most appropriate and convenient option and ensures maximum variation in the sample [48]. The focus group was recruited in person during nursing consultations at the primary care center to ensure that long-stay immigrants had enough experience with PC nurses. Nurses were informed of the project and the inclusion criteria by a senior researcher. Recruitment was difficult; it took two months to conduct the focus group, so we decided to switch strategies and conduct the semi-structured interviews to explore the emerging issues in greater depth, with the first author and observer of the focus group recruiting patients from the focus group. Only two accepted, and they were the ones who had been in Spain for the shortest amount of time and had university degrees. Therefore, in the third phase of recruitment, cultural mediators—who had been previously informed of the project—were asked to search for long-stay immigrants who had been in Spain for over 15 years and had no university studies. In-person recruitment continued until data saturation was established by “repeated responses” in the interview of each participant [49]. All long-stay immigrants who participated were given a complimentary card to cover travel expenses. 

### 2.5. Data Collection

From May 2016 to January 2017, a focus group and five semi-structured interviews were carried out. The number of focus group participants was originally 10 immigrant patients but two of them could not participate in the end due to personal reasons. The focus group was conducted by J.S.-B. and one observer, F.R.-R., both of whom were trained in qualitative methods. Neither worked at the Maresme Primary Care Center so they had not previously met the study participants. The meeting was held at the PC center so the setting would feel familiar, accessible, and safe to participants—this being an important requirement for open and sincere discussion [50]. The meeting lasted approximately one and a half hours and was audio-recorded. The observer took field notes during the focus group. The initial script for the focus group was developed according to the a priori objectives and assumptions of the researchers. The form and content of the script was validated using the Delphi technique, which evaluates the coverage of the questions based on the research objectives, the cognitive complexity and linguistic adequacy of the questions, and the degree to which the items were consistent with the objectives [51]. In this sense, a group of five experts (three nurses experienced in primary care and multiculturality, one of them a daughter of immigrants, and two academic nurses, experts in qualitative methodology) was asked to review the script. Two consultation rounds were carried out asynchronously to organize ideas and make decisions based on the experts’ opinions, finally arriving at a consensus on the final script in the third and last round [51,52].

The script consisted of six issues: general health-related topics, interaction with nurses, interactions and incidents, communication expectations, communication skills, and cultural knowledge. It began by exploring general health-related topics so the group would feel at ease and then went deeper into the study topics. The questions asked to guide the focus group were: “We would like to know about your relationship with your nurse. Please tell us about any positive or negative experiences you have had with her.”; “Tell us about the type of communication between you and your nurse. Is it what you expected?”; “Tell us what type of problems prompted you to visit the nurse, how the nurse helped you resolve them, and whether you are satisfied.”; “Give us an example of how the nurses show that they are motivated to better understand the problems that affect you.” 

After analyzing the focus group, we wanted to know whether participants would maintain the discourse on both the issues included in the script and the emerging issues (no importance given to nurses’ cultural knowledge, and expectations of more humanity in communication) on an individual basis. Therefore, individual, semi-structured interviews were scheduled. These interviews followed a structure similar to that of the focus group, with the addition of the emerging issues. The script was validated by two experts in qualitative methods and multiculturalism. The questions asked to guide the interviews were: “Tell me about some positive experiences from nursing visits; that is, what is done well and you wouldn’t change. And in your opinion, what is done badly and what you would improve?”; “What is missing for you in the nursing visit? How do you feel in the nurse’s office? Tell me about your expectations and whether or not they were met”; “Explain your relationship with your nurse. Describe how the nurse shows respect for your opinion, culture, and customs.” FR conducted semi-structured interviews. The interviews lasted 45–70 min and were audio-recorded.

### 2.6. Data Analysis

The interviews were analyzed following Charmaz’s approach to grounded theory analysis [53]. All recordings were transcribed verbatim and were then reviewed prior to coding. Alongside the data collection process (interviews and focus groups), the first author wrote memos on the preliminary analysis and focused on data processing. Initially, the first author engaged in open coding, that is, line-by-line coding using ATLAS.ti, scientific software for qualitative data analysis. Codes were based on the data and consisted mainly of in vivo codes labeled as “long-stay immigrants” [43]. Next, in a focused, selected phase, the most significant and/or frequent open codes were selected [53] and, depending on the content, were grouped into more general codes, thus establishing the analytical categories. The entire analysis was conducted following the constant comparison method [54] in order to establish analytical distinctions and then make comparisons at each level of the analysis. Each coding phase was discussed with the second author, and all discrepancies were resolved by consensus. Finally, a third researcher—who had not previously participated—reviewed both the findings and the coding process, and a registered nurse reviewed the findings.

### 2.7. Rigor

To reinforce the credibility of the study, a preliminary draft of the paper was submitted to one participant for feedback. To reinforce confirmability, two researchers, who were also experts in qualitative methods, conducted an independent analysis. The findings were also discussed with a researcher who did not participate in the interviews or the analysis. In order to reduce any confirmation bias that might arise in the researchers’ feedback, results were also discussed with a nurse practitioner. Analysis of and participant quotations illustrating main themes contributed to the consistency of the findings [55].

## 3. Results

The focus group consisted of eight long-stay immigrants, with a mean age of 42 and a mean length of residence in Spain of 13 years; 50% were women. Five long-stay immigrants were interviewed, with a mean age of 48 and a mean length of residence of 20 years; 60% were women. 

The findings were grouped into five categories: downplaying the importance of culture, communication with more humanity and respect, communication as a tool, power imbalance in communication, and defending the nurses and avoiding conflict. 

### 3.1. Downplaying the Importance of Culture

The issue of nurses’ cultural knowledge did not come up spontaneously in immigrants’ discourse since the priority for most of them was being heard and having their health concerns well understood. The majority of participants downplayed the importance of cultural issues to the extent that they did not believe it was necessary to take the patient’s culture into account to provide good care, as expressed by one Gambian man who arrived to Spain at the age of 20:

“Culture has nothing to do with good work and care.”

(P5 I)

According to the patients, the only cultural differences that the nurses detected and respected were related to Ramadan. During assessment and dietary prescription, nurses did respect and take into consideration Muslim patients and the restrictions of Ramadan, including adjustments to care plans. However, they did not pay attention to the different eating habits of patients from other cultures.

“They are not interested in the culture of women from Morocco or South America. What they focus on is your health and how to help you. Except for Ramadan, when they ask things they need to know.”

(P2 I)

According to participants the nurses did not consider their treatment preferences either. More often than not, a standard treatment was imposed when patients asked for alternatives, including natural or non-pharmacological therapies. Patients want their perspective to be taken into consideration, especially when they’re interested in therapeutic alternatives. For example, one 49-year-old Latina woman with a university degree mentioned that in some cultures, treatment preferences, such as for natural therapies or other non-pharmacological treatments, differ from those offered by the local healthcare system. Healthcare professionals do not even take those into account, while from the point of view of immigrants, they might be useful in solving their health issues.

“You trust more classic pharmacopoeia over natural remedies. You consider that other stuff witchcraft, shamanism. A Latino person tends to rely more on natural remedies.”

(P6 FG-P4 I)

In addition, some women highlighted professionals’ disregard of gender issues, such as the embarrassment they feel about being seen naked and/or being examined by men. One Latina woman, aged 49, described feeling very uncomfortable when a male nurse had to help her and saw her half-naked.

“Latina women are modest about some things. We want female nurses, not male nurses (...) You have to know who you’re dealing with, be tactful, and ask.”

(P2 I)

Despite not recognizing culture as a modulating element in their relationship with nurses, the healthcare culture of the host country did affect participants. Many followed the care protocols for chronic illnesses even when they did not agree with the diagnosis: they adhered to the health protocol of the host culture, if only partially. For example, one 51-year-old Ecuadorian man living in Spain for over 14 years was diagnosed with hypertension and needed treatment. He refused treatment because he did not believe he was ill; he felt fine and therefore did not agree with the diagnosis. Nonetheless, he continued attending the follow-up visits established in the nursing protocol to monitor and control hypertension.

“The nurse says I have a problem: high blood pressure. But I do not think I have that problem, and I tell her I do not want to take meds. Then, she knows I am not taking them, she has marked that in the record (...) And I continue going to the follow-up visits.”

(P2 FG)

### 3.2. Communication with More Humanity and Respect

The participants appreciated nurses who were cordial, kind, and who showed an interest in their health problems, as well as their needs, family, and environment. They all referred to the nurses using the feminine gender as their experience had been with female nurses. They believed that healthcare providers should treat them in an appropriate, polite, pleasant and professional way. They appreciated closeness, support, and the reassurance the nurse transmitted, both with her words and her attitude.

“When you see a nurse taking care of you with genuine interest, you like it. Then, you leave the visit with a good impression.”

(P2 FG)

In fact, some immigrant patients describe very positive encounters with nurses. One 49-year-old Peruvian woman with a university degree who had been living in Spain for nine years, expressed gratitude for the welcoming attitude of the nurse who accompanied her in what she considered as a difficult time for her and her son: he had anxiety issues that made everyday life hard.

“The nurse was very supportive to me and my son; she was really sensitive. That is greatly appreciated when a child has anxiety issues (…). Sometimes you need a hug, and when a nurse says a kind word, it’s like a hug, some support in the midst of the fear.”

(P6 FG-P4 I)

However, some long-stay immigrants observed a lack of motivation and commitment in some nurses. They expressed their desire for greater humanity in communication and for nurses to show more patience and sensitivity. Dialogue and listening were considered to be the pillars of good healthcare. They valued kind gestures, such as a smile, and believed that certain skills are essential in human interaction.

“An essential skill for interacting with people is a sensitive attitude: having a bit of empathy and knowing how to give emotional support.”

(P4 GF)

“Smiling and being nice doesn’t cost a thing.”

(P7 GF)

### 3.3. Communication as a Tool

Participants consider open communication a tool for negotiation and establishing an honest therapeutic relationship. Nevertheless, they point to the fact that many nurses focus on the specific problem that brought the patient to the clinic, and the majority calls for a more holistic approach, with more time to listen and understand what the patient has to say and joint planning of the strategies aimed at solving his/her health problems. They also reported having many questions to which they often get no answer or are not even able to ask. One man (P1 I) and woman (P4 I) put it this way:

“I talk a lot and ask many questions; I am very persistent (...). And I want to know about my problem, what is going on in my body, why I am like this, and how am I going to get better.”

(P6FG-P4 I)

“I want to speak freely to be able to describe my problem in detail. And always be able to ask.”

(P1 I)

Immigrant patients request a lot of information about their health condition, details about the care plan, the reasons for referral to other services, types of tests performed and why they are being requested, etc. Most wanted to collaborate in their treatment and care plan but were not able to do so. In their opinion, providers should consider the way they would like to be cared for to reach agreements after open discussion. One 34-year-old Maghrebi woman with coeliac disease told us about her experience. The doctor referred her to a nurse to learn more about following a gluten-free diet. She felt worried and disappointed when the nurse simply gave her a piece of paper with information taken from the internet, without specifically discussing her problem or asking her hardly any questions, without probing what she already knew, what she wanted to know or what her routines were, and without asking her anything to adapt the care plan to her needs.

“When we explain our problems to the nurse, she only focuses on the problem. She prints out a sheet of paper and gives it to us. But she should ask people what is wrong, how they feel and how they want to be treated.”

(P7 FG)

Similarly, one 37-year-old Pakistani man with a university degree believed that when being referred to a nurse for medical treatment, care should not be limited to a medical prescription. Rather, the specific problem that brought the patient to the clinic should be assessed and patients should be fully engaged in their own health care.

“Ask patients about their needs, how they want to solve the issue, and then you can come to a common agreement. Communication is a very good bridge.”

(P3 FG)

For long-stay immigrants, communication is the tool required to express their needs, participate and resolve their health issues. However, they acknowledge that the limited amount of time nurses are allowed for visits is a barrier to the relationship. It does not enable them to open an effective communication channel to express the context of their consultation or their needs and doubts, and it limits the bidirectional flow of information. Moreover, they find it prevents nurses from listening actively and, as a result, patients feel poorly understood. This is how one Peruvian woman described it in the focus group:

“Nurses don’t have time to listen because they’re completely focused on doing their thing, you know? Then, there won’t be any communication (...). I want the nurse to listen to me, to take time to care for me and let me to explain how I feel.”

(P6 FG-P4 I)

### 3.4. Power Imbalance in Communication

In general, participants expressed that some healthcare providers adopt an imposing, authoritarian attitude that triggers feelings of inferiority and a sense of blind obedience. One Latino man living in Spain for over 14 years explained that he thinks healthcare professionals keep control in encounters from a privileged position of power. He often perceives little interest in and indifference towards his needs and opinions. He puts it this way:

“They shouldn’t take their position to heart. Because it’s often: 'I’m here to work.' And I feel this power and authority. And you, there, don’t matter to them. But if they stop that way of thinking and act in a more sensitive way, the patient is going to be very happy with that person.”

(P2 GF)

Immigrants identified prejudiced attitudes in some professionals who treated them inappropriately, showing disdain, indifference and ignoring them. On occasions, they even make reference to certain acts of covert racism when they did not feel they were being treated as they should be and associated such treatment to their status as foreigners. The same Peruvian woman who previously demanded to be heard and allowed time to explain how she felt, reported that, on some occasions, she felt she was treated with disdain for being a foreigner, which made her feel inferior and angry.

“Sometimes they treat you badly. At first, you try to make excuses for her (...). But then you start thinking she treats you like that because you are a spic and she doesn’t like you (...). I feel powerless; I feel angry, but I swallow my anger.”

(P4 I- P6 FG)

One 48-year-old Moroccan man who immigrated at the age of 18 explained that he often had problems with his nurse outside the office. She treated him with indifference and did not respond to his questions if he asked them before entering the office. Once inside, he felt that she went more quickly than with other patients because he was an immigrant.

“If you are a foreigner, then you enter through one door and go straight out another.”

(P1 GF)

The relationship with health professionals is established or influenced based on past experiences, while present experiences modulate already existing images of the nurse. Study participants have accumulated many experiences after residing in the host country for years. This is the case of one African woman living in Catalonia for 16 years, married to a Catalan man and now fluent in Catalan and Spanish. She explains how mistreated she felt while in labor with her only child, shortly after arriving to Catalonia. Lacking knowledge about how to navigate the health system, barely speaking the language and about to give birth, she was initially refused admission. She was admitted in the end at the insistence of her Catalan husband, although she felt the treatment she received was not appropriate at all.

“I didn’t speak the language and didn’t understand a thing (…) The nurses said, ‘Go away, leave, this is not the place to give birth!’ And I wanted to have the child; I was in labor (…). But in the end, they took me in. Like crap: ‘Wash up! (…)’. African women give birth in a tree.”

(P4 FG)

She describes her experience, explaining the prejudice and racism she perceived from the professionals. In the long run, this perception of an unequal relationship distances the immigrant patients from the specific professionals involved, as well as from other professionals and the healthcare system in general. For example, one Moroccan woman who had been in the country for 10 years reported that, despite changing nurses, she still kept her distance from the new one given her previous bad experience.

“I didn’t like her arguing with me so I didn’t come back again. I have another nurse now, but I still keep away.”

(P7 FG)

Faced with this unequal relationship, patients demanded compassionate, dignified, polite, professional and prejudice-free treatment. In addition, they rejected controversy, arguments, and bad manners because they promote distance and distrust in the health staff and the healthcare system. 

### 3.5. Defending the Nurses and Avoiding Conflict

When talking about unpleasant experiences, most of the immigrants spoke with a hint of empathy and acknowledgement: they tended to understand the nurses, recognizing their efforts toward dialogue, and to avoid confrontation. This is very well represented in the story of one 34-year-old Maghrebi woman.

“Maybe that lady was feeling bad, had family problems, or something else (...). And I arrived at the wrong time. One has to try and understand others before judging them.”

(P7 FG)

Some immigrants expressed feelings of remorse after having accepted or excused bad experiences with certain healthcare professionals. They admitted to accepting some things and even excusing or trying to understand the nurses’ or other professionals’ reactions as the fee for their access to the Catalan healthcare system. The vast majority of immigrant patients highlighted the opportunity of universal access to the Catalan public healthcare system—which they believe offers high quality care—as a compelling reason to overlook an unpleasant experience.

“It’s a privilege to have free health care. In our country, you have to pay for insurance. It’s very expensive and this is a luxury for me.”

(P2 FG)

## 4. Discussion

The results show that long-stay immigrants would like closer and more personalized communication with greater humanity and polite and respectful manners. While they do not believe that culture is important in communication, they do perceive signs of an asymmetrical care relationship. Both results are likely applicable to the general population and lead us to suggest that the acculturation process has had an influence on the participants’ responses. This study aimed to explore how long-stay immigrants experience and perceive intercultural communication in encounters with primary care nurses.

### 4.1. The Effect of Acculturation

We expected cultural knowledge to be more relevant from the patient’s perspective, as scholars often emphasize the acquisition of cultural knowledge [56]. However, linguistically competent long-stay immigrants with long acculturation processes do not consider communication problems with nurses to be rooted in culture. These results are consistent with those reported in a qualitative study which also ruled out the cultural aspect in favor of respectful, sensitive and functional communication [57]. Acculturation is a multivariate process of psychological, cultural, and social adaptation in cultural groups that come into direct, continuous contact with another culture [58] and it can lead to changes in values, beliefs, and attitudes in matters of health [25]. Living between cultures, immigrant patients find themselves in continuous negotiation between their values and the values, attitudes, and behaviors of the host country’s healthcare culture. The results of this study reveal adaptation when long-stay immigrants do not verbalize the cultural roots of communication challenges with nurses but rather call for more humanity in communication. Degrie et al. already pointed out that living between cultures requires a balance of different cultural contexts of care [19]. But it must be noted that acculturation associated with the healthcare system, and the relationship with nurses and other healthcare professionals, is characterized by a double vulnerability: that of being a patient and that of being an immigrant from a poor country. Acculturation processes are ingrained in power structures that have an impact on them, and this includes both healthcare culture and relationships with health professionals. On the one hand, as patients, acculturation takes place in a very hierarchical and vertical healthcare system, in which the patient has no knowledge and, therefore, no power. On the other hand, as immigrants from poor countries, social acculturation takes place in a highly disadvantageous economic and political context. These different but interlinked social positions can give rise to different subjective experiences [59].

### 4.2. Humanity in Communication

The findings show that long-stay immigrant patients place great interest in communication competence during nursing visits. Participants expressed that polite and respectful care, active listening and a clear exchange of information were most valuable to them. Patients prefer good technical skills, positive attitudes, effective communication, and relationships that include connection and continuity [60]. Paternotte et al. connects intercultural communication with person-centered communication [22]. She suggests adapting the communication style of professionals to the individual preferences of each patient by developing a set of generic and intercultural communication skills related to language, notions of health and illness, a social perspective of communication and prejudice. She considers it an effective tool for equitable information exchanges, creating space for understanding and participation, thus improving the quality of communication and addressing immigrants’ biopsychosocial needs [22,61]. A literature review on hospital intercultural encounters highlights humanity in care and communication as an influencing factor [19]. Immigrant inpatients look for meaningful care encounters in which they feel respected and cared for as unique human beings, and they point to the relational importance of communication. Interviewees, who had been in the host country for six to 30 years, reported that being well informed allowed them to become important actors of their own lives [30]. The literature has also defined what good communication in health means for certain cultural groups. Latinos, for example, emphasize personal relationships and value sympathy, the expression of feelings, and personalized and friendly relationships [62], while South Asian immigrants value a respectful relationship based on trust that is inclusive of their opinions [63].

### 4.3. Rebalancing the Care Relationship

The description of strained relationships, patients’ exoneration of the nurse practitioner’s attitude and conflict avoidance seem to reflect the power imbalance in the nurse-immigrant patient relationship. The literature also shows implicit biases among health professionals towards ethnic minorities [64], for instance, prejudices and stereotypes held by Spanish nurses and conveyed in the form of rejection and power imbalances in intercultural communication with Moroccan patients, and greater discrimination against Moroccan and Romanian populations, as well as African men [65,66]. Despite the prejudices expressed by healthcare staff, the participants of the study downplayed these biased attitudes as they feared losing the high-quality care received and the universal access to healthcare [67]. We must highlight that immigrant patients also hold prejudices or stereotypes about nurses and professionals from the host country, since acculturation works in both directions. Stigmatization, marginalization and alienation are negative preconceptions ingrained not only in nurses and other professionals, but also in immigrant patients, making both patients and healthcare professionals look at each other as the “other” instead of “one of us” [30]. Otherness is a reciprocal phenomenon in cross-cultural encounters between patient and healthcare professional that results in mistrust [30]. When otherness is accompanied by the fact that all patient-professional relationships are more or less asymmetric (epistemically asymmetric, given that one part has skills and knowledge that the other part does not have and needs) [30] and the fact that they take place in a very hierarchical healthcare environment defined by authoritarian and paternalistic attitudes, what follows is that patients’ autonomy is undermined and mistrust and insecurity increase. In Western healthcare systems, the hierarchical medical model predominates. It is defined by an asymmetric power relationship that is reinforced during intercultural communication, thus promoting the perpetuation of imposed norms of thought, behavior and interaction [68]. The social positions of patient and immigrant, which already establish a power imbalance on their own, are simultaneously intertwined with other social traits such as gender, age and class, thus shaping the asymmetric relationship (Arora et al 2019). Several authors touch on the concept of intersectionality, explaining that an individual’s various social positions mold his/her experience [59], in this case, of communication with the nurse, while other social positions such as ethnicity, gender, age and social standing are simultaneous categories of oppression that constitute power imbalances [59,69].

#### Culturally Safe Care

The literature highlights that treating all patients in the same way is associated with culturally unsafe practices that do not respond to the specific health needs of immigrant populations [70]. However, there have also been studies showing that this lack of safety goes beyond culture: the power imbalance and lack of patient-centered communication, motivation and commitment are expressions of culturally “unsafe” health care [71]. A clinical ethnographic study of Australian aboriginal patients suggests that active listening together with patient-centered communication—featuring more dialogue, a bidirectional flow and more information—has the potential for culturally safe care [72]. To Canadian aboriginal patients, positive and culturally safe experiences include the expression of feelings and compassionate interaction between persons [70]. Cultural safety entails a process of self-reflection by professionals on power imbalance and cultural imposition, changes in attitudes and behaviors, the development of communication skills, and the creation of an atmosphere of respect, where concepts and knowledge can be shared and where there is mutual collaboration and learning [71]. Other authors, while not using the term “culturally safe,” state that care is expressed through listening and conversation, mutual respect, trust, and the confidence provided by the therapeutic relationship [60]. It should be noted that the same attributes identified in “safe experiences” of healthcare (dialogic, bidirectional communication, active listening, and recognizing values and beliefs) are not limited by culture or language but by unequal power relationships inherent to healthcare culture.

### 4.4. Health Communication and Gender

Study participants complained that nurses offered standardized solutions that do not respond to the care request. Our results were sometimes conditioned by issues related to gender, such as women’s feelings of shame when parts of their body are exposed to or examined by men. A recent literature review shows how preconceived notions of embarrassment among women from different cultures complicates care and health examinations [41]. This means that the availability and gender of the health provider is important, especially among South Asian, Chinese and Muslim women. Immigrant women prefer women health professionals for their care in general but especially for reproductive health and physical examinations [41,59]. The literature shows that women health professionals have a more personal, emotional and fair communication style [73]. In this study, participants were mostly in contact with female nurses. Study participants appreciated female nurses who were cordial, kind, and who showed an interest in their health problems. The female dyad (female patient-female professional) is described in the literature as more patient-centered and as having longer consultations conducted in an atmosphere of ease and equality [74], although asymmetrical relationships have also been described in such encounters [73].

### 4.5. Limitations

The small sample size of this study does not allow for generalizability. Nonetheless, it is the first of its kind in Spain and one of the few examples in the literature about the intercultural communication of long-stay immigrants with long acculturation processes in encounters with primary care nurses. The results are presented as a first step towards future in-depth research. The study has some other limitations, as well. The purposive sampling was guided by very specific criteria on language fluency, such that people not fluent in the local language were excluded despite their potential vulnerability to miscommunication. Although we acknowledge that there may be selection bias, by selecting immigrant patients fluent in Spanish, language posed a less serious communication obstacle (widely described in the literature) and the exploration of non-language-related cultural and communicative aspects was encouraged, which adds to the strength of the study. Finally, we only explored the perspectives of long-stay immigrants. Comparison with non-immigrant patients and an evaluation of healthcare provider perspectives would be necessary to better understand the dynamics of these relationships in context. However, our results offer insight into an area where evidence is scarce, and they raise new issues that deserve further investigation.

### 4.6. Implications

The results of this study may be of help to nurses, as well as other healthcare professionals, administrations, policy makers and researchers in designing specific programs adapted to long-stay immigrants’ needs and challenges (re-balancing the therapeutic relationship and adding humanity to communication).

#### 4.6.1. Public Health Implications

The results suggest that the acculturation process that long-stay immigrants undergo might shape experiences similar to those of native Spaniards. Given that power imbalance is the result of an unequal relationship that also exists in the case of nationals, linguistically competent long-stay immigrants should be included as stakeholders in the different participatory bodies within the healthcare system so that their voices and needs are heard. This strategy would promote the creation of an atmosphere of respect, mutual cooperation and learning and represent a step toward cultural safety [31]. Therefore, health institutions should elaborate protocols, procedures and policies together with different cultural communities so that when a long-stay immigrant patient enters the health system they can be automatically applied. A process of self-reflection and awareness of the privileged position of nurses is essential to promote actions aimed at re-balancing power. Failure to engage in such processes may lead to situations of inequality among long-stay immigrants that could have been avoided. 

#### 4.6.2. Practical Implications

The study results suggest the need for critical intervention in clinical practice in order to ensure culturally safe relationships with long-stay immigrants who have undergone a process of acculturation. Such intervention must be geared towards improving communication and power inequality in care encounters. For that to happen, professionals need to acknowledge the effect of acculturation in long-stay immigrants and understand communication as a tool for negotiation in therapeutic relationships, leaving prejudices and previous experiences behind. Nursing practitioners’ education should be adapted to the specific needs of long-stay immigrants. It is essential to provide effective communication training to health professionals [40]. When professionals offer direct and honest information, the asymmetry in their relationships with patients is reduced [29]. Nurses should be trained in person-centered intercultural communication so that (a) they can improve their overall communication skills, (b) increase their cultural awareness to avoid prejudice, and (c) include immigrant patients in the decision-making process. Because care expectations differ according to gender, such training should take a gender-sensitive approach that includes this aspect in all levels of health communication [73]. Long-stay immigrants could participate as storytellers in such training, given the effectiveness of such narratives in health sciences degrees [75,76]. We must take into account the perspective and experiences of long-stay immigrants if we want to move the system forward through more holistic and biopsychosocial approaches.

#### 4.6.3. Implications for Research

A deeper understanding of the complex mechanisms involved in the communication between professionals and long-stay immigrant patients is crucial. Further research with larger sample sizes that takes into account the point of view and narratives of long-stay immigrant is needed. To better understand the dynamics of the relationship between long-stay immigrants and nurses in context, comparison is also needed with: (a) non-immigrant patients, (b) healthcare provider perspectives and (c) newly arrived immigrant patients, thus allowing for deeper understanding of the changes resulting from acculturation. Moreover, an intersectional approach would be useful to examine health communication by placing attention on how intersections of age, socio-economic status and gender influence immigrants’ daily lives and encounters in healthcare settings [77]. 

## 5. Conclusions

Despite its limitations, this study contributes to the limited selection of literature that takes into account the perspective of long-stay immigrants with long acculturation processes on intercultural communication with primary care nurses. The findings show that long-stay immigrants in our setting face several communication barriers, including professionals’ failure to listen and a strained relationship with healthcare providers that indicates a power imbalance between themselves and nurses. They also reveal immigrants’ tendency to make excuses for professionals to avoid conflict. This suggests that immigrant patients do not feel the environment is culturally safe, which violates the concept of equitable health care.

Identifying the elements that facilitate intercultural communications with long-stay immigrant patients offers an opportunity to detect and modify inappropriate and insensitive care so that adapted care, both in communicative content and form, may be provided instead. This would take us closer to a horizontal care relationship. Nurses should be trained in person-centered intercultural communication, although such training would not be effective if structural changes focused on insufficient and overstretched staff do not take place as well. A process of self-reflection, awareness-raising and commitment is also necessary, both at the personal and institutional level, to eliminate the asymmetry in the nurse-patient relationship. It is the nurses’ responsibility at the micro-level and the responsibility of institutions and policies at the macro-level to make effective intercultural communication a reality. The commitment to provide fair, inclusive and deliberate care that is sensitive to the needs of long-stay immigrants must form part of the political agenda. We are aware that a structural shift in power relationships is needed in the healthcare system. Achieving effective intercultural communication and cultural safety requires a process of self-reflection, awareness-raising and commitment, both at a personal and institutional level, to eliminate the asymmetry in the nurse-patient relationship.

## Figures and Tables

**Table 1:** Characteristics of focus group participants.

Participant	Sex	Age	Country of origin	Years in Spain	Education Level
P1	Man	48	Morocco	30	Primary school
P2	Man	51	Ecuador	14	Secondary school
P3 *	Man	37	Pakistan	6	University
P4	Woman	41	Ivory Coast	16	Secondary school
P5	Man	37	Senegal	9	No schooling
P6 *	Woman	49	Peru	9	University
P7	Woman	34	Morocco	10	Secondary school
P8	Woman	38	China	12	Secondary school

* Also participated in the in-depth interviews.

**Table 2:** Characteristics of interview participants.

Participant	Sex	Age	Country of origin	Years in Spain	Education level
P1 *	Man	37	Pakistan	6	University
P2	Woman	45	Morocco	15	Secondary school
P3	Woman	48	Gambia	29	Primary school
P4 *	Woman	49	Peru	9	University
P5	Man	61	Gambia	41	Primary school

* Also participated in the focus group.

## Data Availability

Data sharing is not applicable to this article. The interviews content or transcriptions are not available for public archiving or sharing because it was not included in participants’ consent. Use of data was only for study purpose and its specific conditions regarding confidentiality, privacy protection and data handling was approved by Ethical and Clinical Investigation Committee of the Primary Care Research Institute IDIAP Jordi Gol, and it was described to participants upon recruitment.
